# Antidepressants and Road Safety: A Forensic Toxicological Perspective Based on Observational Data and Current Evidence

**DOI:** 10.3390/ph19071118

**Published:** 2026-07-20

**Authors:** Davide Filardi, Francesca Vernich, Federico Mineo, Giulio Mannocchi, Roberta Tittarelli

**Affiliations:** 1 Laboratory of Forensic Toxicology, Section of Legal Medicine, Social Security and Forensic Toxicology, Department of Biomedicine and Prevention, Faculty of Medicine and Surgery, University of Rome Tor Vergata, Via Montpellier 1, 00133 Rome, Italy; davide.filardi@students.uniroma2.eu (D.F.); francesca.vernich@uniroma2.it (F.V.); f.mineo@med.uniroma2.it (F.M.); giulio.mannocchi@uniroma2.it (G.M.); 2 PhD School in Medical-Surgical Applied Sciences, University of Rome Tor Vergata, Via Montpellier 1, 00133 Rome, Italy

**Keywords:** antidepressants, driving fitness, psychomotor impairment, forensic toxicology, road safety

## Abstract

**Background/Objectives:** Antidepressants are widely prescribed medications that may affect psychomotor performance and driving ability depending on their pharmacological profile. This study investigated the prevalence and patterns of antidepressant use among drivers undergoing forensic toxicological evaluation and explored their potential implications for road safety. **Methods:** An observational study was conducted on *n* = 6316 drivers undergoing forensic toxicological assessment following licence suspension for driving under the influence (DUI) of alcohol and/or drugs between January 2023 and December 2025. Reported antidepressants were classified into selective serotonin reuptake inhibitors (SSRIs), serotonin–norepinephrine reuptake inhibitors (SNRIs), serotonin antagonist and reuptake inhibitors (SARIs), tricyclic antidepressants (TCAs), norepinephrine–dopamine reuptake inhibitors (NDRIs), noradrenergic and specific serotonergic antidepressants (NaSSAs), and monoamine oxidase inhibitors (MAOIs). Distribution patterns were analysed descriptively and discussed considering the available literature. **Results:** Antidepressant use was reported by *n* = 132 participants (2.1%). SSRIs were the most common class (44.0%), followed by SNRIs (26.5%) and SARIs (17.4%). TCAs (6.1%), NDRIs (3.7%), and NaSSAs (2.3%) were less common, while no MAOI use was reported. Among antidepressant users, *n* = 28 individuals (21.2%) tested positive for other psychoactive substances, including benzodiazepines, cocaine, and cannabinoids. **Conclusions:** The use of antidepressants was relatively uncommon in the study population. However, clinical evaluation remains important, particularly at the start of treatment and during dose adjustments to determine the potential for an increased risk while driving. Further studies integrating toxicological analyses and clinical data are needed to better define the relationship between antidepressant exposure, polysubstance use, and road safety.

## 1. Introduction

**Figure 1:**
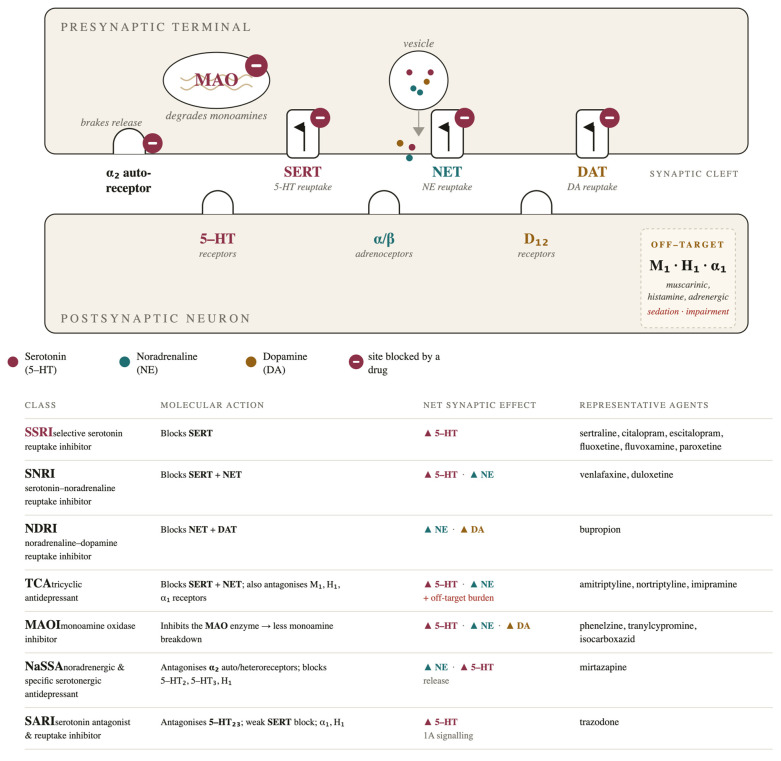
Schematic representation of the principal mechanisms of action of antidepressant classes and their effects on monoaminergic neurotransmission.

Depression is one of the most prevalent mental disorders worldwide and represents a major public health concern due to its profound impact on individual functioning, social participation, and quality of life. According to the World Health Organization (WHO), depression is projected to become the leading cause of disability globally by 2030, highlighting the growing burden of mood disorders on health systems and society [1]. The increasing recognition and diagnosis of depressive disorders have been accompanied by a substantial rise in pharmacological treatments, particularly antidepressant medications. Antidepressants are a heterogeneous class of psychopharmacological agents primarily indicated for major depressive disorder but also commonly prescribed for anxiety disorders, obsessive–compulsive disorder, and other psychiatric conditions [2,3]. Their therapeutic effects are mediated through the modulation of central neurotransmitter systems, including serotonin, norepinephrine, and dopamine, which play a crucial role in mood regulation, cognition, and psychomotor control. Figure 1 provides an overview of the principal molecular targets and mechanisms of action underlying the pharmacological heterogeneity of antidepressants. As antidepressant use has become increasingly common among active drivers, concerns have emerged regarding their potential impact on road safety. Both depressive symptoms and antidepressant-related adverse effects may impair cognitive, attentional, and psychomotor functions essential for safe driving. Over the past two decades, antidepressants have become among the most frequently prescribed medications in Europe and other high-income regions. This increase is attributable not only to the growing recognition of depressive and anxiety disorders, but also to improved access to mental health care through community-based services and primary care integration that have facilitated earlier diagnosis and broader treatment availability. The progressive reduction in social stigma has also increased acceptance of both psychiatric disorders and antidepressant therapy among clinicians and patients.

In Italy, data from the Italian Medicines Agency (AIFA) have shown a steady increase in antidepressant consumption. In 2021, approximately 7% of the population reported using antidepressant medications, an increase of 2.4 percentage points compared with the previous year [4]. This trend has been further influenced by the COVID-19 pandemic, which was associated with a marked rise in the prevalence of depressive and anxiety symptoms across many countries [5].

Safe driving requires intact cognitive, attentional, and psychomotor functions. While effective treatment of depression may improve concentration and reduce accident risk by alleviating core symptoms, several antidepressants, particularly those with sedative or anticholinergic properties, have been associated with transient impairments in alertness, reaction time, and motor coordination, especially early in treatment or after dose adjustments [6,7]. Epidemiological and experimental studies [7,8,9] have shown that antidepressants may exert heterogeneous effects on driving-related abilities, depending on pharmacological class, dosage, treatment duration, and individual patient characteristics. Adverse effects such as sedation, daytime sleepiness, reduced attention, impaired reaction time, blurred vision, dizziness, and psychomotor slowing, may negatively affect fitness to drive. Conversely, effective treatment of depressive symptoms may improve cognitive functioning, concentration, decision-making, and overall driving performance in patients whose underlying psychiatric condition itself impairs safe driving ability [7]. Some studies have reported that tricyclic antidepressants (TCAs) and other marked sedative or anticholinergic properties may acutely impair driving-related abilities, manifesting as drowsiness, reduced alertness, delayed reaction times, and psychomotor slowing [8]. In contrast, newer antidepressants, especially selective serotonin reuptake inhibitors (SSRIs) and serotonin–norepinephrine reuptake inhibitors (SNRIs), generally display a more favourable safety profile and are not routinely associated, in prescribing information, with clinically significant driving impairment [10,11,12]. The contribution of controlled substances, including prescription medications and drugs of abuse, to road traffic accidents is increasingly recognized, with a growing number of road crashes being associated with their consumption [13].

However, regulatory approaches and clinical recommendations regarding antidepressant use and driving vary considerably across countries. In Italy, the impact of antidepressant medications on driving ability has received limited attention within traffic safety legislation, and antidepressant use is currently not subject to specific regulatory restrictions or mandatory medical–legal assessment with respect to fitness to drive.

The aim of the present study was to investigate the prevalence and patterns of antidepressant use among drivers undergoing forensic toxicological assessment following licence suspension, while providing a comprehensive narrative review of the current evidence regarding the effects of antidepressants on psychomotor performance and driving ability. The novelty of this work lies in the integration of real-world observational data obtained from a forensic cohort undergoing medico-legal fitness-to-drive evaluation with a critical analysis of the available literature. This combined clinical and forensic approach provides a broader perspective on the potential implications of antidepressant use for road safety.

### 1.1. Antidepressants and Driving Safety

#### 1.1.1. Selective Serotonin Reuptake Inhibitors (SSRIs)

The most commonly prescribed SSRIs include sertraline, citalopram, escitalopram, fluoxetine, fluvoxamine, and paroxetine [14].

In addition to major depressive disorder, SSRIs are widely prescribed for anxiety disorders, obsessive–compulsive disorder, and other psychiatric conditions. Overall, SSRIs are generally well tolerated, exhibiting minimal sedative effects and a relatively low risk of impairing driving performance compared with other antidepressant classes [15].

Abrupt discontinuation of serotonin reuptake inhibitors may lead to psychological and somatic withdrawal symptoms, ranging from mild manifestations to more severe reactions, including delirium in rare cases [16]. Fluoxetine has been associated with higher rates of weight loss, agitation, and anxiety than other SSRIs [17].

Fluvoxamine is also available in oral formulations for the treatment of major depressive disorder and obsessive–compulsive disorder. Compared with other SSRIs, it has been associated with a higher incidence of somnolence and sedation, particularly during the initial phase of treatment, although controlled studies suggest only limited impairment of psychomotor performance overall and no relevant effects on driving-related skills in healthy volunteers [18]. Consistently, pharmacological data indicate that fluvoxamine at therapeutic doses has no, or negligible, influence on the ability to drive or use machines; however, somnolence has been reported in clinical use, and caution is therefore recommended until the individual response to treatment has been established [19].

Paroxetine is used for the treatment of major depressive episodes, obsessive–compulsive disorder (OCD), panic disorder (with or without agoraphobia), social anxiety disorder/social phobia, generalised anxiety disorder and post-traumatic stress disorder. Paroxetine exhibits a favourable tolerability profile but has a higher tendency to induce sedation, especially during early treatment [17]. Its shorter half-life and stronger anticholinergic effects increase the risk of drowsiness and impaired concentration, particularly in older adults. Clinical studies show that paroxetine can slow reaction times and reduce attention, key factors for safe driving. Epidemiological evidence indicates an increased risk of road traffic accidents during the first weeks of therapy [13]. Although all SSRIs may cause mild sedative effects, such as somnolence, fatigue, dizziness, or impaired concentration, they are generally considered relatively safe with respect to driving performance compared with other antidepressant classes. Nevertheless, caution is recommended when driving or operating machinery, particularly during treatment initiation and following dose adjustments.

#### 1.1.2. Serotonin Norepinephrine Reuptake Inhibitors (SNRIs)

The two SNRIs most frequently prescribed by medical practitioners are venlafaxine and duloxetine. Venlafaxine is widely used in clinical practice; however, approximately 5% of patients may experience adverse effects of considerable severity. Abrupt discontinuation of treatment has been associated with potentially severe withdrawal symptoms, often manifesting within hours of the last dose. Such symptoms can substantially impair motor functions and coordination, highlighting the importance of strict adherence to the prescribed regimen and refraining from activities that could compromise safety, such as driving [20].

As a class, SNRIs generally show limited impact on driving performance, although mild somnolence, dizziness, and fatigue may occur, particularly during treatment initiation [9,21].

Withdrawal symptoms associated with venlafaxine include dizziness, anxiety, and paraesthesia, particularly following abrupt discontinuation. Given its half-life of approximately four hours (ten hours for the active metabolite), even short delays in administration may trigger withdrawal effects. Gradual discontinuation over 7–10 days is recommended to mitigate these risks [22].

Duloxetine, another SNRI, is employed in the treatment of depression, anxiety, and chronic pain. Studies have demonstrated that it can enhance cognitive functions, including psychomotor speed and attention, independently of mood improvement [23]. Nevertheless, drowsiness, dizziness, fatigue, and balance disturbances may occur, especially during the initial weeks of treatment, potentially affecting the ability to drive safely [23]. While no studies specifically address duloxetine’s effects on driving in real-world settings, patients should exercise caution. Abrupt discontinuation or overdose may also pose clinically significant risks, as reported for venlafaxine.

Recent pharmacovigilance analyses have highlighted clinically relevant adverse effects associated with milnacipran and levomilnacipran, including psychiatric and cardiovascular symptoms, which may be more pronounced with levomilnacipran because of its stronger noradrenergic activity. To the best of our knowledge, no studies have evaluated the effects of these agents on driving performance or real-world driving. Nevertheless, they warrant consideration because they belong to the SNRI class and remain widely prescribed, particularly in the United States [24].

#### 1.1.3. Norepinephrine–Dopamine Reuptake Inhibitors (NDRIs)

NDRIs represent a class of antidepressants that selectively inhibit the reuptake of norepinephrine and dopamine. Bupropion is the main representative of this class, approved for the treatment of major depressive disorders and as an aid for smoking cessation. Compared with other antidepressants, bupropion is characterized by its stimulant properties, minimal sedative effects, and a lower risk of weight gain [25].

Studies show that therapeutic doses of bupropion do not significantly impair reaction time, oculo-manual coordination, or divided attention, suggesting that the drug is generally safe for activities requiring complex psychomotor performance, such as driving or operating machinery [26].

NDRIs are generally associated with low sedative effects and a limited risk of psychomotor impairment, making them among the antidepressants least likely to adversely affect driving ability [27].

#### 1.1.4. Tricyclic Antidepressants (TCAs)

TCAs, including amitriptyline and nortriptyline are used in the treatment of depression and remain widely prescribed for chronic pain, insomnia, and mood disorders. Their pharmacological profile is characterised by pronounced anticholinergic and antihistamine effects, resulting in substantial sedation. Common adverse effects include drowsiness, slowed reflexes, blurred vision, and impaired motor coordination, all of which may significantly compromise driving ability [13].

Amitriptyline has been shown to impair driving performance. In a simulator study involving healthy volunteers, a single dose reduced road-tracking ability and vigilance, with maximal effects observed approximately four hours after administration [28]. Conversely, long-term users of sedative antidepressants such as amitriptyline (treatment > 3 years) did not demonstrate clinically significant impairment in on-road driving.

Although imipramine and nortriptyline are generally less sedating than amitriptyline, they share anticholinergic properties and require caution, particularly during treatment or following dose adjustments [29].

Overall, available evidence from clinical studies and regulatory safety assessments indicates that tricyclic antidepressants as a class may impair alertness, psychomotor performance and driving ability. Although the magnitude of these effects varies across individual agents, including clomipramine, nortriptyline, desipramine, doxepin, trimipramine, dosulepin, lofepramine, and protriptyline, a clinically relevant anticholinergic and sedative burden is consistently observed. Consequently, caution is warranted when these medications are used in activities such as driving [15,30].

#### 1.1.5. Monoamine Oxidase Inhibitors (MAOIs)

MAOIs, including phenelzine, tranylcypromine, and isocarboxazid, represent a historical class of antidepressants used for the treatment of atypical or treatment-resistant depression. Although currently prescribed less frequently than other antidepressant classes, MAOIs remain an important therapeutic option in selected subjects with treatment-resistant depression [27]. Their use has gradually declined due to dietary restrictions and the potential for significant drug interactions. MAOIs act by inhibiting monoamine catabolism, which strongly affects central neurotransmission and may influence psychomotor function [31].

Common adverse effects of monoamine oxidase inhibitors include dizziness, hypotension, sedation, and insomnia, all of which may negatively affect driving safety, particularly during treatment or following dose adjustments.

Patients prescribed MAOIs should be counselled on potential psychomotor impairment, and extra caution is warranted when engaging in activities such as driving, particularly during the early phase of treatment or following dose adjustments.

#### 1.1.6. Noradrenergic and Specific Serotonergic Antidepressants (NaSSAs)

Mirtazapine, the main NaSSA, is prescribed for major depressive disorder, anxiety disorders, and eating disorders such as anorexia nervosa and bulimia. Its efficacy in depression is comparable to other antidepressants, with benefits for patients experiencing comorbid anxiety or sleep disturbances [32]. Mirtazapine has a favourable safety profile and sustained therapeutic effect, supporting its use in long-term treatment [33].

Studies reported that administration of mirtazapine for one to two weeks can improve psychomotor performance and simulated driving abilities in patients with depression, reflecting functional recovery alongside symptom reduction [34]. Single doses, particularly when taken at bedtime, may transiently impair driving, but these effects diminish with continued use. Individual risk assessment is recommended during the early stages of treatment to ensure safe driving.

#### 1.1.7. Serotonin Antagonists and Reuptake Inhibitors (SARIs)

SARIs constitute a distinct class of antidepressants, with trazodone being the most widely prescribed medication [35]. Trazodone is commonly used in the treatment of major depressive disorder and, due to its pronounced sedative properties, is frequently prescribed for insomnia at low to moderate doses. Despite its sedative profile, available evidence suggests a favourable safety profile with respect to cognitive and psychomotor functions [36].

Experimental studies conducted in healthy volunteers have shown that repeated administration of trazodone at therapeutic doses does not significantly impair driving performance, attention, or cognitive function when compared with placebo. No clinically relevant effects on reaction time, vigilance, or motor coordination were observed, supporting its tolerability in activities requiring sustained attention and psychomotor efficiency [35]. Nevertheless, transient sedation and residual drowsiness may occur during treatment, warranting caution in individual patients, especially when trazodone is administered in combination with other central nervous system depressants.

Consequently, SARIs may reduce psychomotor performance in some individuals, particularly during treatment initiation or when combined with other central nervous system depressants [35].

#### 1.1.8. Off-Label Pharmacological Strategies in Depressive Disorders

Off-label pharmacotherapy is increasingly employed in treatment-resistant mood disorders, particularly when conventional antidepressant regimens fail to achieve remission or when clinical phenotypes present atypical characteristics requiring alternative neuropharmacological modulation [37]. Several agents with established psychotropic properties are used based on available clinical evidence and individualized benefit–risk assessment. Among these agents, lamotrigine has demonstrated efficacy in bipolar depression, particularly in preventing depressive relapse, and is often used off-label in treatment-resistant presentations [38]. Valproate is commonly used as an adjunct in affective disorders characterized by irritability, mood lability, and impulsivity, while carbamazepine has a more historical role due to metabolic interactions and safety concerns [39].

Gabapentin and pregabalin, although not antidepressants per se, are prescribed in depressive conditions with comorbid anxiety, neuropathic pain, or somatic distress, where symptom relief may indirectly improve emotional regulation; however, clinical evidence supporting their efficacy is limited [37]. Quetiapine, an atypical antipsychotic, shows robust evidence for adjunctive use in both unipolar and bipolar depression and is recommended as a second-line augmentation strategy for patients with major depressive disorder who do not respond adequately to initial antidepressant therapy [40].

From a forensic toxicological perspective, the sedative and psychomotor-impairing properties of these agents, including dose-dependent drowsiness associated with gabapentinoids, cognitive slowing attributable to quetiapine, and the potential for residual sedation with lamotrigine at higher doses, may compromise driving ability to a degree comparable with, or exceeding, that of conventional antidepressants. Consequently, clinicians who prescribe off-label agents to patients who drive should exercise the same caution warranted for first-line antidepressant classes, particularly during treatment initiation and dose-escalation phases.

## 2. Results

Between January 2023 and December 2025, a total of n = 6316 subjects underwent toxicological tests, distributed annually as follows: n = 2474 cases in 2023, n = 1811 in 2024, and n = 2031 in 2025. Most participants were male (5446; 86.2%), whereas females accounted for 870 (13.8%). The overall mean age was 41 years (range 18–85 years).

**Figure 2:**
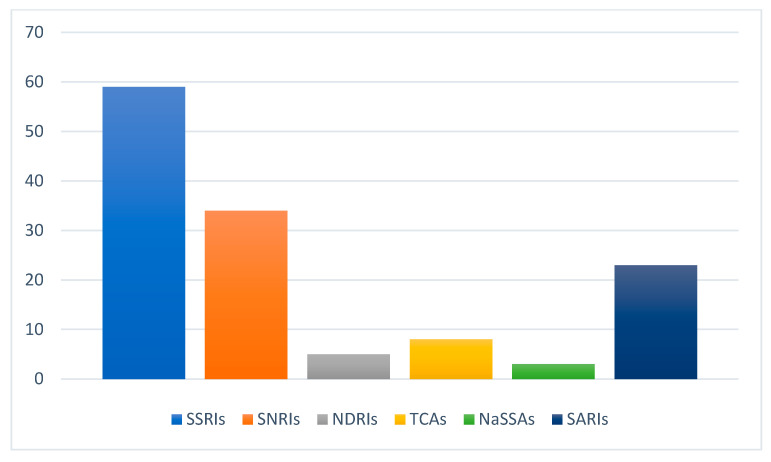
Total number of reported antidepressants divided per class.

During the study period, n = 132 subjects (2.1% of the total sample) reported antidepressant use. The most frequently declared pharmacological classes were SSRIs (n = 58; 44.0%), SNRIs (n = 35; 26.5%), and SARIs (n = 23; 17.4%). Less commonly reported classes included TCAs (n = 8; 6.1%), NDRIs (n = 5; 3.7%), and NaSSAs (n = 3; 2.3%). No use of MAOIs was reported (Figure 2).

Among SSRIs, paroxetine (n = 20) and sertraline (n = 18) were the most frequently reported, while escitalopram (n = 8), fluoxetine (n = 7), and citalopram (n = 5) were less commonly declared. Within SNRIs, duloxetine (n = 18) and venlafaxine (n = 17) were reported with similar frequency.

Other drugs included bupropion (NDRI, n = 5), amitriptyline and clomipramine (TCAs, n = 8), mirtazapine (NaSSA, n = 3), and trazodone (SARI, n = 23). These results highlight both the predominance of SSRIs and the therapeutic diversity within pharmacological classes.

**Figure 3:**
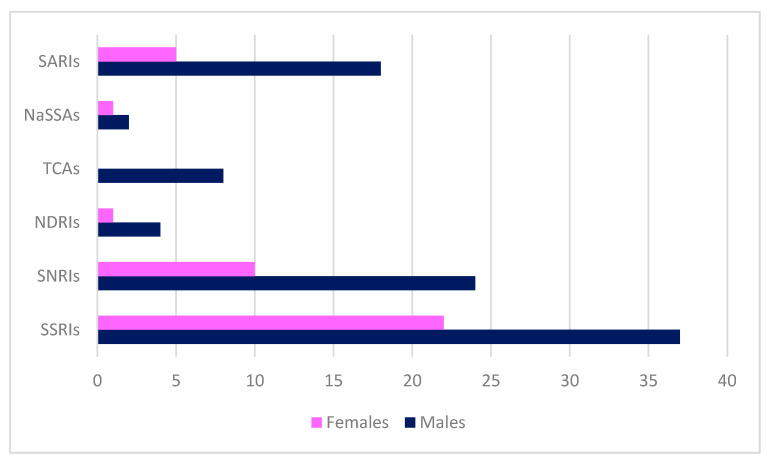
Distribution of antidepressant users by pharmacological class and gender.

Overall, antidepressant use was more common among males (n = 93; 70.5%) than females (n = 39; 29.5%), consistent with the sex distribution of the study population. However, SSRIs and SNRIs showed a relatively more balanced sex distribution compared with TCAs and SARIs. Within the subgroup of antidepressant users, the mean age was 45 years for both males and females (Figure 3).

## 3. Discussion

The pattern of antidepressant use observed in our study appears broadly consistent with contemporary Italian and European prescribing trends. According to data from the AIFA [4], approximately 7% of the Italian population received antidepressant treatment in 2021, with SSRIs representing the most frequently prescribed antidepressant class. Similarly, a recent European analysis of antidepressant consumption across 25 countries reported that SSRIs remain the dominant antidepressant class throughout Europe [41], whereas tricyclic antidepressants and MAOIs are prescribed considerably less frequently, as also observed in our study. In our cohort, SSRIs represented the most frequently reported antidepressants (44.0%). Although the overall prevalence of self-reported antidepressant use in our population (2.1%) was substantially lower than estimates reported for the Italian general population, the relative distribution of antidepressant classes appears to reflect current prescribing practices observed in both Italy and other European countries. The lower prevalence observed in our study should be interpreted with caution, as antidepressant use was based exclusively on self-report and the study population consisted of a highly selected group of drivers undergoing mandatory forensic toxicological assessment.

**Figure 4:**
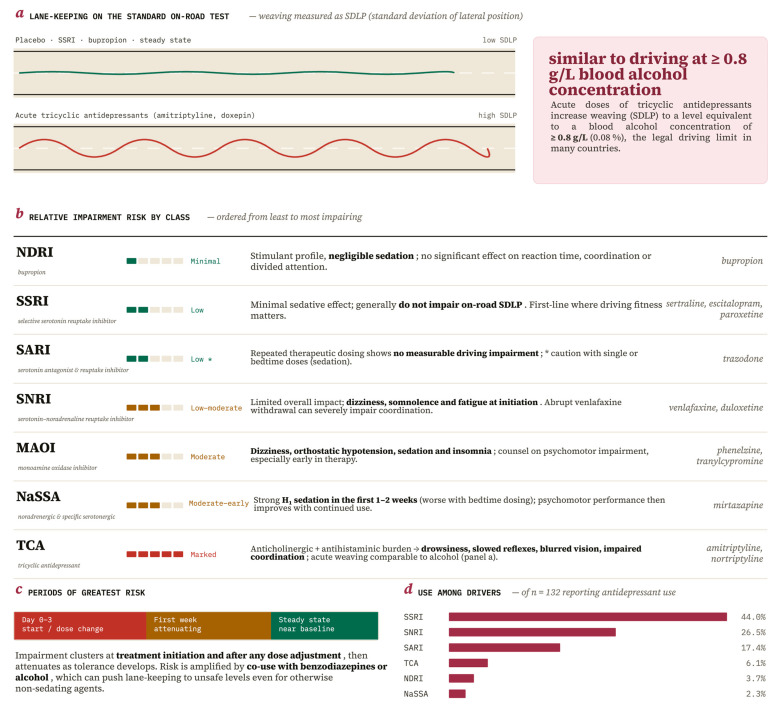
Summary of the relative driving impairment risk associated with the main antidepressant classes based on the literature reviewed. Panels (**a**–**c**) summarize evidence from experimental and clinical studies regarding the potential effects of antidepressant classes on driving performance, psychomotor function, and periods of increased impairment risk. Panel (**d**) reports the distribution of antidepressant classes among drivers included in the present observational forensic cohort (n = 132).

Current evidence indicates marked differences among antidepressant classes in their effects on psychomotor performance and driving ability (Figure 4). Panels a,b compares the relative impact of the main antidepressant classes on driving performance, identifying tricyclic antidepressants as those associated with the greatest impairment. Panels c,d depict the temporal profile of impairment together with the distribution of antidepressant classes within the forensic toxicological cohort.

Although antidepressant use is generally more common among women in the general population, most antidepressant users in our cohort were male (70.5%). This finding likely reflects the marked predominance of men in the overall study population, where males accounted for 86.2% of all subjects undergoing forensic toxicological evaluation following licence suspension. Therefore, the higher proportion of male antidepressant users should be interpreted within the context of the specific forensic population investigated and should not be considered indicative of a higher prevalence of antidepressant use among males. Rather, it reflects the demographic characteristics of drivers referred for medico-legal assessment. Consequently, sex-specific findings should be interpreted with caution and cannot be directly generalized to the general population.

Current evidence suggests that driving-related risks may arise not only during treatment initiation and dose escalation but also during abrupt discontinuation or rapid tapering of antidepressants, particularly short half-life agents, owing to the occurrence of withdrawal symptoms that may adversely affect psychomotor performance and fitness to drive [42]. It should also be emphasized that the present study cannot distinguish the effects of antidepressant therapy from those of the underlying depressive disorder, as no information regarding psychiatric diagnosis or untreated depression was available in this cohort. This distinction is clinically relevant because depression itself may impair attention, psychomotor speed, and decision-making, thereby increasing crash risk, whereas successful antidepressant treatment may improve these functions by alleviating depressive symptoms. Previous studies have shown that patients receiving stable long-term SSRI/SNRI treatment may exhibit driving performance comparable to healthy controls, while untreated depression has been associated with impaired driving ability and an increased risk of road traffic accidents [6,43].

Furthermore, antidepressants do not uniformly impair driving ability, with effects varying by class, dose, treatment phase, concomitant medications, and underlying condition. Impairment appears greater with sedating agents and during treatment initiation or dose escalation, whereas SSRIs are generally associated with a lower impact on psychomotor performance and driving ability [44].

All samples were analysed for alcohol biomarkers. Additional analyses were performed on different biological matrices, including urine, blood, and keratin samples. Confirmatory testing was carried out using liquid chromatography–tandem mass spectrometry (LC–MS/MS) [45,46,47], gas chromatography–mass spectrometry (GC–MS) [48] and GC-MS/MS [49,50,51], in accordance with current forensic guidelines [52,53]. Alcohol misuse was further assessed through established biomarkers, including serum carbohydrate-deficient transferrin (CDT), routinely used for the identification of chronic heavy drinking patterns [54] and ethyl glucuronide (EtG) in hair, which provides objective evidence of long-term alcohol consumption [55].

Toxicological analyses of the entire cohort identified a subgroup of 28 individuals who reported antidepressant use and simultaneously tested positive for other psychoactive substances. This subgroup comprised 21 males and 7 females, with a mean age of 44 years. Detected substances included benzodiazepines, cocaine, cannabinoids, and barbiturates.

Regarding alcohol-related biomarkers, all subjects included in the present analysis tested negative for CDT. However, interpretation was inconclusive in two cases because of the presence of genetic transferrin variants, which are known to interfere with CDT quantification and may preclude definitive result interpretation [56]. Among the subset of subjects who underwent EtG hair analysis, all samples tested negative, providing no evidence of chronic alcohol consumption. In particular, the presence of cannabinoids and different benzodiazepines, including lorazepam, nordiazepam, oxazepam, temazepam, triazolam, and alprazolam, was detected in subjects who reported the use of SSRIs, such as paroxetine and sertraline. Alprazolam was also identified in subjects reporting the use of SNRIs, including venlafaxine, as well as SARIs, represented by trazodone.

Urinary toxicological screening revealed cocaine positivity in some subjects, indicating concomitant cocaine use among individuals reporting paroxetine treatment.

Cocaine was detected in both urine and hair samples from subjects who declared the use of paroxetine, sertraline, and trazodone. Cannabinoids were identified in subjects reporting the use of both SSRIs and SARIs.

Furthermore, the concomitant use of multiple benzodiazepines in combination with antidepressant therapy was observed in both male and female subjects, suggesting a relevant pattern of simultaneous exposure to central nervous system depressants. Such associations are of particular concern in the context of driving safety, given the potential additive or synergistic effects on psychomotor performance, attention, and reaction time.

In addition, codeine, oxycodone, and benzodiazepines were detected in urine samples from subjects reporting sertraline use.

Only one subject tested positive for phenobarbital while reporting trazodone use.

Overall, these findings highlight the clinical and forensic relevance of polydrug use involving antidepressants, drugs of abuse, and other central nervous system depressants.

Drug interactions may influence driving ability through both pharmacokinetic and pharmacodynamic mechanisms rather than by simple additive effects. For example, some SSRIs inhibit cytochrome P450 enzymes involved in benzodiazepine metabolism, potentially increasing benzodiazepine exposure and enhancing central nervous system depression [57]. Likewise, concomitant use of serotonergic antidepressants and cocaine may produce complex monoaminergic interactions, and rare cases of serotonin syndrome have been reported [58]. Therefore, driving impairment should be assessed on an individual basis. Although a preliminary classification of driving risk (e.g., low, moderate, or high) might be clinically useful, the available evidence and the descriptive nature of our study do not support such a simplified approach, as risk depends on multiple factors including antidepressant class, dose, treatment phase, individual susceptibility, and concomitant use of alcohol or other psychoactive substances.

The concomitant use of cocaine and antidepressants observed in the present cohort is consistent with previous reports describing the frequent coexistence of depressive disorders and cocaine use disorder. Antidepressant treatment is commonly prescribed in this population, and the simultaneous exposure to antidepressants and cocaine has been documented in both clinical and experimental settings [59]. These findings further support the need for careful clinical and forensic evaluation of polysubstance use when assessing fitness to drive.

### Limitations

Our study has several limitations that should be acknowledged. Antidepressant use was based exclusively on self-report obtained during clinical history taking and was not analytically confirmed in biological matrices such as blood, urine, or hair. This reflects routine clinical practice, as testing for antidepressants is not part of the toxicological screening protocol required by the Local Medical Commission (CML) for fitness-to-drive assessments. Consequently, the reported prevalence may have been affected by both underreporting and overreporting. Furthermore, the study population consisted exclusively of drivers undergoing mandatory forensic toxicological assessment following licence suspension, which may limit the generalizability of these findings to the broader driving population. In addition, the marked imbalance in sex distribution within the cohort represents a limitation, as it prevents reliable evaluation of sex-specific patterns of antidepressant use and precludes direct comparison with epidemiological data from the general population. Information on antidepressant dosage, treatment duration, adherence, and timing of administration was also unavailable, precluding a more detailed evaluation of exposure-related effects. Finally, the observational and descriptive nature of the study does not allow causal inferences regarding the relationship between antidepressant use and driving impairment.

## 4. Materials and Methods

This study was designed as an original observational investigation based on real-world forensic toxicology data collected from drivers undergoing mandatory fitness-to-drive assessment. The observational component was complemented by a narrative review of the current literature to provide clinical and pharmacological context. These two components were intended to be complementary: the observational data describe real-world patterns of antidepressant use in a forensic driving population, whereas the literature review supports the interpretation of their potential implications for psychomotor performance and road safety.

A narrative literature search was performed using electronic databases, including PubMed and Google Scholar, up to December 2025. The search strategy was designed to identify relevant publications evaluating the effects of antidepressants on driving performance, psychomotor function, and road safety by combining controlled vocabulary terms and free-text keywords. Search terms included combinations of keywords such as “antidepressants and driving performance,” “SSRIs and driving ability,” “SNRIs and road safety,” “TCAs and traffic accidents,” and “antidepressants and driving ability.” Additional relevant studies were identified by screening the reference lists of selected articles. Observational data were collected from drivers undergoing toxicological assessment following convictions for driving under the influence of alcohol or drugs. The assessment was mandated by the CML to determine medical fitness to drive.

All subjects underwent forensic toxicological evaluation according to standardized protocols for the assessment of fitness to drive after violations of Articles 186 (driving under the influence of alcohol), 186-bis (applicable to novice and professional drivers), and 187 (driving under the influence of drugs) of the Italian Highway Code. Eligible participants were aged ≥18 years. The study population therefore represents the complete cohort of subjects referred to the Forensic Toxicology Laboratory of the University of Rome Tor Vergata during the study period. No additional inclusion or exclusion criteria were applied, and no subjects were excluded because of refusal to undergo toxicological testing, as participation in the forensic assessment is part of the mandatory medico-legal procedure. Biological specimens, including urine, blood, and keratin matrices, were analysed according to established forensic toxicology procedures and in accordance with national and international guidelines for medico-legal and forensic drug testing [52,53].

The toxicological investigations included the analysis of drugs of abuse and biomarkers of alcohol consumption. Alcohol assessment comprised the determination of CDT and, when indicated, EtG in hair as markers of chronic alcohol exposure. Analyses were carried out using chromatographic techniques coupled with mass spectrometric detection including LC–MS/MS, GC-MS/MS.

Data reported in this study were collected from 1 January 2023 to 31 December 2025. Information on antidepressant use was obtained from standardized anonymous questionnaires completed by participants during the forensic medical evaluation.

Data extracted from the questionnaires were processed and graphically represented using Microsoft Excel^®^ 2016 (MSO 16.0.4738.1000, Microsoft Corporation, Redmond, WA, USA). Descriptive statistics were used to summarize demographic characteristics and patterns of antidepressant use. Only descriptive statistics were performed due to the observational design and limited sample size, which did not allow reliable inferential analyses.

## 5. Conclusions

The findings of the present study, together with the available evidence from the literature, suggest that greater clinical attention should be given to antidepressant use in the context of driving. In Italy, such medications are not currently regulated with respect to fitness to drive, despite evidence suggesting that some antidepressants, particularly during treatment initiation or dose adjustments, may affect psychomotor performance in specific clinical conditions. Antidepressants are not included in the schedules of the Italian Presidential Decree No. 309/1990 (DPR 309/90), which regulates narcotic and psychotropic substances and identifies those subject to specific control measures. As a result, antidepressants are generally not regarded as substances requiring specific regulatory monitoring in relation to driving fitness. In light of the available evidence, greater attention to antidepressant use during fitness-to-drive assessments may be warranted. Further research is needed to evaluate whether specific clinical or medico-legal approaches could improve risk assessment and road safety.

In this cohort of drivers undergoing mandatory forensic toxicological assessment, antidepressant use was relatively uncommon, and the distribution of pharmacological classes was consistent with European prescribing patterns. However, the interpretation of these findings is limited by the self-reported nature of antidepressant use and the selected population, which may limit the generalisability of these findings to the broader driving population.

The observed concomitant use of antidepressants with benzodiazepines, cocaine, and other psychoactive substances further highlights the clinical and forensic relevance of polydrug exposure. From a pharmacokinetic and pharmacodynamic perspective, the concomitant use of SSRIs or SNRIs with benzodiazepines may be associated with additive or potentially synergistic effects on central nervous system depression, leading to greater impairment of vigilance, reaction time, and divided attention than either drug alone. Conversely, concurrent cocaine use may paradoxically reduce the perception of sedation while maintaining or potentially increasing psychomotor impairment. This pattern is of forensic relevance when assessing fitness to drive, as affected individuals may underestimate their actual level of impairment.

Future studies should integrate analytical confirmation of antidepressant exposure with epidemiological and experimental approaches to better characterise their real-world impact on driving performance.

A multidisciplinary approach involving clinicians, toxicologists, and regulatory authorities may help support evidence-based policies, improve patient counselling, and strengthen risk assessment strategies. The present observational findings suggest that antidepressant use alone is not necessarily associated with increased driving risk. However, concomitant use with benzodiazepines, alcohol, opioids, or illicit stimulants such as cocaine may represent a critical factor for potential psychomotor impairment. Given the descriptive nature of the present study, these observations should be interpreted cautiously and should not be regarded as evidence of causal relationships. Further prospective studies are warranted to better define the relationship between antidepressant use, polysubstance exposure, and road safety.

## Data Availability

The data presented in this study were obtained from the included studies and are openly available.

## Abbreviations

The following abbreviations are used in this manuscript:

SSRIs	Selective Serotonin Reuptake Inhibitors
SNRIs	Serotonin–Norepinephrine Reuptake Inhibitors
NDRIs	Norepinephrine–Dopamine Reuptake Inhibitors
SARIs	Serotonin Antagonists and Reuptake Inhibitors
NaSSAs	Noradrenergic and Specific Serotonergic Antidepressants
MAOIs	Monoamine Oxidase Inhibitors

## References

[1] World Health Organization (2017). Depression and Other Common Mental Disorders: Global Health Estimates.

[2] Szuhany K.L., Simon N.M. (2022). Anxiety Disorders: A Review. JAMA.

[3] OECD (2022). European Union Health at a Glance: Europe 2022: State of Health in the EU Cycle.

[4] Italian Medicines Agency (AIFA) (2022). National Report on Medicines Use in Italy—OsMed 2021.

[5] OECD (2021). Tackling the Mental Health Impact of the COVID-19 Crisis: An Integrated, Whole-of-Society Response.

[6] Hill L.L., Lauzon V.L., Winbrock E.L., Li G., Chihuri S., Lee K.C. (2017). Depression, Antidepressants and Driving Safety. Inj. Epidemiol..

[7] Brunnauer A., Laux G. (2017). Driving Under the Influence of Antidepressants: A Systematic Review and Update of the Evidence of Experimental and Controlled Clinical Studies. Pharmacopsychiatry.

[8] Ravera S., Ramaekers J.G., De Jong-van Den Berg L.T.W., De Gier J.J. (2012). Are Selective Serotonin Reuptake Inhibitors Safe for Drivers? What Is the Evidence?. Clin. Ther..

[9] Brunnauer A., Buschert V., Fric M., Distler G., Sander K., Segmiller F., Zwanzger P., Laux G. (2015). Driving Performance and Psychomotor Function in Depressed Patients Treated with Agomelatine or Venlafaxine. Pharmacopsychiatry.

[10] Gutiérrez-Abejón E., Herrera-Gómez F., Criado-Espegel P., Álvarez F.J. (2020). Trends in Antidepressants Use in Spain between 2015 and 2018: Analyses from a Population-Based Registry Study with Reference to Driving. Pharmaceuticals.

[11] Olesen A.V., Madsen T.K.O., Lahrmann H., Nielsen J. (2022). Use of Psychotropic Medication and Risk of Road Traffic Crashes: A Registry-Based Case–Control Study in Denmark, 1996–2018. Psychopharmacology.

[12] Westerhuis F., Van Dijken J.H., Veldstra J.L., Brookhuis K.A., Verster J.C., Van De Loo A.J.A.E., Vinckenbosch F.R.J., Vermeeren A., Van Der Sluiszen N.N.J.J.M., Ramaekers J.G. (2024). Driving Performance of Long-Term Users of Sedating Antidepressants and Benzodiazepines. Traffic Inj. Prev..

[13] Orriols L., Salmi L., Philip P., Moore N., Delorme B., Castot A., Lagarde E. (2009). The Impact of Medicinal Drugs on Traffic Safety: A Systematic Review of Epidemiological Studies. Pharmacoepidemiol. Drug Saf..

[14] Poulsen H., Raymond O., McCarthy M.J. (2025). The Use of Prescription Medication and Other Drugs by New Zealand Drivers with Illegal Blood Alcohol Levels. Traffic Inj. Prev..

[15] Brunnauer A., Laux G., Geiger E., Soyka M., Möller H.-J. (2006). Antidepressants and Driving Ability: Results From a Clinical Study. J. Clin. Psychiatry.

[16] Blum D., Maldonado J., Meyer E., Lansberg M. (2008). Delirium Following Abrupt Discontinuation of Fluoxetine. Clin. Neurol. Neurosurg..

[17] Edinoff A.N., Akuly H.A., Hanna T.A., Ochoa C.O., Patti S.J., Ghaffar Y.A., Kaye A.D., Viswanath O., Urits I., Boyer A.G. (2021). Selective Serotonin Reuptake Inhibitors and Adverse Effects: A Narrative Review. Neurol. Int..

[18] Westenberg H.G.M., Sandner C. (2006). Tolerability and Safety of Fluvoxamine and Other Antidepressants: TOLERABILITY and SAFETY OF FLUVOXAMINE. Int. J. Clin. Pract..

[19] Agenzia Italiana del Farmaco (AIFA) Riassunto delle Caratteristiche del Prodotto—Fluvoxamina. https://www.google.com/url?sa=t&source=web&rct=j&opi=89978449&url=https://api.aifa.gov.it/aifa-bdf-eif-be/1.0.0/organizzazione/1561/farmaci/34669/stampati%3Fts%3DRCP&ved=2ahUKEwjFgejx5K6UAxUs2gIHHTA3HQQQFnoECA0QAQ&usg=AOvVaw2P5Q9W5_dZV1CD86URCsiF.

[20] Campagne D.M. (2005). Venlafaxine and Serious Withdrawal Symptoms: Warning to Drivers. Medscape Gen. Med..

[21] Mandrioli R., Protti M., Mercolini L. (2018). New-Generation, Non-SSRI Antidepressants: Therapeutic Drug Monitoring and Pharmacological Interactions. Part 1: SNRIs, SMSs, SARIs. Curr. Med. Chem..

[22] Ellingrod V.L., Perry P.J. (1994). Venlafaxine: A Heterocyclic Antidepressant. Am. J. Hosp. Pharm..

[23] Greer T.L., Sunderajan P., Grannemann B.D., Kurian B.T., Trivedi M.H. (2014). Does Duloxetine Improve Cognitive Function Independently of Its Antidepressant Effect in Patients with Major Depressive Disorder and Subjective Reports of Cognitive Dysfunction?. Depress. Res. Treat..

[24] Huang T., Zhang P., Zhou Y., Wang L., Zhang Q., Li M., Xiao J. (2025). Disproportionality Analysis of Safety Signals for Milnacipran and Levomilnacipran: A Pharmacovigilance Study Using the FDA Adverse Event Reporting System. Front. Pharmacol..

[25] Papakostas G.I., Stahl S.M., Krishen A., Seifert C.A., Tucker V.L., Goodale E.P., Fava M. (2008). Efficacy of Bupropion and the Selective Serotonin Reuptake Inhibitors in the Treatment of Major Depressive Disorder With High Levels of Anxiety (Anxious Depression): A Pooled Analysis of 10 Studies. J. Clin. Psychiatry.

[26] Siepmann M., Werner K., Schindler C., Oertel R., Kirch W. (2005). The Effects of Bupropion on Cognitive Functions in Healthy Subjects. Psychopharmacology.

[27] Van Den Eynde V., Abdelmoemin W.R., Abraham M.M., Amsterdam J.D., Anderson I.M., Andrade C., Baker G.B., Beekman A.T.F., Berk M., Birkenhäger T.K. (2023). The Prescriber’s Guide to Classic MAO Inhibitors (Phenelzine, Tranylcypromine, Isocarboxazid) for Treatment-Resistant Depression. CNS Spectr..

[28] Iwamoto K., Takahashi M., Nakamura Y., Kawamura Y., Ishihara R., Uchiyama Y., Ebe K., Noda A., Noda Y., Yoshida K. (2008). The Effects of Acute Treatment with Paroxetine, Amitriptyline, and Placebo on Driving Performance and Cognitive Function in Healthy Japanese Subjects: A Double-blind Crossover Trial. Hum. Psychopharmacol..

[29] Brunnauer A., Herpich F., Zwanzger P., Laux G. (2021). Driving Performance Under Treatment of Most Frequently Prescribed Drugs for Mental Disorders: A Systematic Review of Patient Studies. Int. J. Neuropsychopharmacol..

[30] Podewils L.J., Lyketsos C.G. (2002). Tricyclic Antidepressants and Cognitive Decline. Psychosomatics.

[31] Hoffman G.R., Olson M.G., Schoffstall A.M., Estévez R.F., Van Den Eynde V., Gillman P.K., Stabio M.E. (2023). Classics in Chemical Neuroscience: Selegiline, Isocarboxazid, Phenelzine, and Tranylcypromine. ACS Chem. Neurosci..

[32] Verster J.C., Van De Loo A.J.A.E., Roth T. (2015). Mirtazapine as Positive Control Drug in Studies Examining the Effects of Antidepressants on Driving Ability. Eur. J. Pharmacol..

[33] Anttila S.A.K., Leinonen E.V.J. (2001). A Review of the Pharmacological and Clinical Profile of Mirtazapine. CNS Drug Rev..

[34] Brunnauer A., Laux G., David I., Fric M., Hermisson I., Moller H.-J. (2008). The Impact of Reboxetine and Mirtazapine on Driving Simulator Performance and Psychomotor Function in Depressed Patients. J. Clin. Psychiatry.

[35] Sasada K., Iwamoto K., Kawano N., Kohmura K., Yamamoto M., Aleksic B., Ebe K., Noda Y., Ozaki N. (2013). Effects of Repeated Dosing with Mirtazapine, Trazodone, or Placebo on Driving Performance and Cognitive Function in Healthy Volunteers. Hum. Psychopharmacol..

[36] Khouzam H.R. (2017). A Review of Trazodone Use in Psychiatric and Medical Conditions. Postgrad. Med..

[37] Ng Q.X., Han M.X., Teoh S.E., Yaow C.Y.L., Lim Y.L., Chee K.T. (2021). A Systematic Review of the Clinical Use of Gabapentin and Pregabalin in Bipolar Disorder. Pharmaceuticals.

[38] Arnone D., Östlundh L., Mosa M., MacDonald B., Oldershaw J., Qassem T., Young A.H. (2025). Efficacy of Lamotrigine in the Treatment of Unipolar and Bipolar Depression: Meta-Analysis of Acute and Maintenance Randomised Controlled Trials. Pharmaceuticals.

[39] Hui Poon S., Sim K., Baldessarini R.J. (2015). Pharmacological Approaches for Treatment-Resistant Bipolar Disorder. Curr. Neuropharmacol..

[40] Olver J.S., Ignatiadis S., Maruff P., Burrows G.D., Norman T.R. (2008). Quetiapine Augmentation in Depressed Patients with Partial Response to Antidepressants. Hum. Psychopharmacol..

[41] Bindel L.J., Seifert R. (2026). Antidepressant Drug Use in Europe: Past Consumption, Prescribing Patterns and Forecast until 2030. Int. J. Clin. Pharm..

[42] Wilson E., Lader M. (2015). A Review of the Management of Antidepressant Discontinuation Symptoms. Ther. Adv. Psychopharmacol..

[43] Van Der Sluiszen N.N.J.J.M., Vermeeren A., Verster J.C., Van De Loo A.J.A.E., Van Dijken J.H., Veldstra J.L., Brookhuis K.A., De Waard D., Ramaekers J.G. (2019). Driving Performance and Neurocognitive Skills of Long-term Users of Benzodiazepine Anxiolytics and Hypnotics. Hum. Psychopharmacol..

[44] Sansone R.A., Sansone L.A. (2009). Driving on Antidepressants: Cruising for a Crash?. Psychiatry.

[45] Fisichella M., Morini L., Sempio C., Groppi A. (2014). Validation of a Multi-Analyte LC–MS/MS Method for Screening and Quantification of 87 Psychoactive Drugs and Their Metabolites in Hair. Anal. Bioanal. Chem..

[46] Montesano C., Sergi M., Odoardi S., Simeoni M.C., Compagnone D., Curini R. (2014). A μ-SPE Procedure for the Determination of Cannabinoids and Their Metabolites in Urine by LC–MS/MS. J. Pharm. Biomed. Anal..

[47] Marchei E., Ferri M.A., Torrens M., Farré M., Pacifici R., Pichini S., Pellegrini M. (2021). Ultra-High Performance Liquid Chromatography-High Resolution Mass Spectrometry and High-Sensitivity Gas Chromatography-Mass Spectrometry Screening of Classic Drugs and New Psychoactive Substances and Metabolites in Urine of Consumers. Int. J. Mol. Sci..

[48] Goldberger B.A., Chronister C.W., Merves M.L., Garg U., Hammett-Stabler C.A. (2010). Quantitation of Benzodiazepines in Blood and Urine Using Gas Chromatography-Mass Spectrometry (GC-MS). Clinical Applications of Mass Spectrometry.

[49] Strano-Rossi S., Bermejo A.M., De La Torre X., Botrè F. (2011). Fast GC-MS Method for the Simultaneous Screening of THC-COOH, Cocaine, Opiates and Analogues Including Buprenorphine and Fentanyl, and Their Metabolites in Urine. Anal. Bioanal. Chem..

[50] Tittarelli R., Filardi D., Mineo F., Mannocchi G. (2025). A Novel and Validated GC-MS/MS Method for the Detection of Four Opioids and Seven Fentanoids in Oral Fluid for Forensic Applications. Molecules.

[51] Di Trana A., Sprega G., Kobidze G., Taoussi O., Lo Faro A.F., Bambagiotti G., Montanari E., Fede M.S., Carlier J., Tini A. (2024). QuEChERS Extraction and Simultaneous Quantification in GC-MS/MS of Hexahydrocannabinol Epimers and Their Metabolites in Whole Blood, Urine, and Oral Fluid. Molecules.

[52] Favretto D., Cooper G., Andraus M., Sporkert F., Agius R., Appenzeller B., Baumgartner M., Binz T., Cirimele V., Kronstrand R. (2023). The Society of Hair Testing Consensus on General Recommendations for Hair Testing and Drugs of Abuse Testing in Hair. Drug Test. Anal..

[53] Strano Rossi S., Frison G., Chericoni S., Bertol E., Favretto D., Pichini S., Salomone A., Tagliaro F., Vignali C. (2023). Linee Guida per La Determinazione Di Sostanze Stupefacenti e Psicotrope Su Campioni Biologici Con Finalità Tossicologico-Forensi e Medico-Legali. Riv. Ital. Med. Lab.

[54] Fiorelli D., Romani L., Treglia M., Pallocci M., Passalacqua P., Coppeta L., Marsella L.T., Tittarelli R. (2023). Carbohydrate-Deficient Transferrin (CDT) as a Biomarker of Alcohol Abuse: A Retrospective Study of the Italian Drinking Trend among Drivers from 2016 to 2022. Toxics.

[55] Romani L., Mannocchi G., Mineo F., Vernich F., Stefani L., Marsella L.T., Tittarelli R. (2025). Development and Validation of a Fast and Sensitive UPLC-MS/MS Method for Ethyl Glucuronide (EtG) in Hair, Application to Real Cases and Comparison with Carbohydrate-Deficient Transferrin (CDT) in Serum. Int. J. Mol. Sci..

[56] Schellenberg F., Wielders J., Anton R., Bianchi V., Deenmamode J., Weykamp C., Whitfield J., Jeppsson J.-O., Helander A. (2017). IFCC Approved HPLC Reference Measurement Procedure for the Alcohol Consumption Biomarker Carbohydrate-Deficient Transferrin (CDT): Its Validation and Use. Clin. Chim. Acta.

[57] Sproule B.A., Naranjo C.A., Bremner K.E., Hassan P.C. (1997). Selective Serotonin Reuptake Inhibitors and CNS Drug Interactions: A Critical Review of the Evidence. Clin. Pharmacokinet..

[58] Malik H.U.-R., Kumar K. (2012). Serotonin Syndrome with Escitolapram and Concomitant Use of Cocaine: A Case Report. Clin. Med. Insights Case Rep..

[59] Chalmé R.L., Rubin E., Evans S.M., Haney M., Foltin R.W. (2025). Venlafaxine Treatment Is Associated with Improved Mood, but Not Decreased Cocaine Self-Administration, in Depressed People Who Use Cocaine. Pharmacol. Biochem. Behav..

